# Reflect, Grow, Connect: A Pilot Study on the Potential Benefits of Facilitated Group Supervision for Radiation Therapists

**DOI:** 10.1002/jmrs.70043

**Published:** 2025-12-17

**Authors:** Gay Dungey, Ryan Rodger, Lily Martin

**Affiliations:** ^1^ Department of Radiation Therapy University of Otago Wellington New Zealand; ^2^ Advanced Orthopaedic Physiotherapist and Allied Professions Workforce Development Coordinator Wellington Hospital Wellington New Zealand

## Abstract

**Introduction:**

Facilitated Group Supervision (FGS) involves a group of professionals meeting under the guidance of a supervisor to discuss workplace challenges and promote reflective practice. Previous supervision styles have been trialled for radiation therapists (RTs) in New Zealand with mostly successful results. This pilot study aimed to explore how RTs perceived FGS and compare this with their experiences of previous supervision models.

**Methods:**

This mixed‐methods pilot study was conducted at the Wellington Blood and Cancer Centre with seven RTs. Participants met in two groups every four weeks for six months with an independent allied health‐trained facilitator. Afterwards, they completed a QUALTRICS questionnaire, including the Clinical Supervision Evaluation Questionnaire, which is 14 Likert scale statements, assessing group process, purpose, and impact. Open‐ended questions gathered qualitative data.

**Results:**

All seven radiation therapists completed the questionnaire, with both qualitative and quantitative results indicating highly positive feedback regarding FGS. Thematic analysis of the qualitative data revealed that participants developed valuable insights and coping strategies, felt the FGS environment enhanced safety and reflection, and found that discussing shared experiences reduced stress. The RTs also preferred FGS to their previous experiences of supervision.

**Conclusion:**

The findings showed a positive perception of FGS among all participating RTs, especially experienced RTs who benefitted from the structure and process. Participants reported gaining valuable insights from both the facilitator and peers, which enhanced their skills and helped address clinical challenges. All participants expressed interest in continuing with FGS, agreed that FGS could benefit all RT professionals, and identified it as their preferred method of supervision.

## Graphical abstract

This mixed‐methods pilot study explored how a small group of New Zealand radiation therapists perceived Facilitated Group Supervision (FGS) compared with previous supervision models. Using questionnaires and thematic analysis after a six‐month intervention, the study found that participants viewed FGS highly positively. They reported enhanced reflection, valuable insights, reduced stress, and a clear preference for adopting FGS as their ongoing supervision approach.

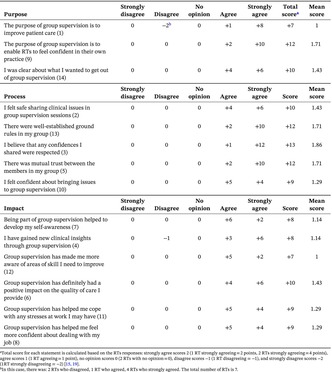

## 1 Introduction

One of the primary challenges facing the radiation therapy (RT) field is low job retention [1, 2], with New Zealand radiation therapists averaging just 8.7 years of service [3]. Research conducted in New Zealand and Australian radiation therapy departments found that the most common reasons for dissatisfaction among radiation therapists were high workloads, challenging working conditions, and limited career progression opportunities [1, 2, 3]. In response to these challenges, supervision has emerged as a structured process aimed at supporting professionals to reflect on their work, refine their practice, enhance well‐being, and improve job retention [4]. Supervision can be conducted in group settings, either Peer Group Supervision (PGS), which is peer‐led [5, 6, 7], or Facilitated Group Supervision (FGS), in which the group is facilitated by a trained professional [8, 9]. Alternatively, it may occur in a one‐on‐one format, known as professional supervision (PS) [10].

Over the past six years, radiation therapists in New Zealand have been trialling supervision. This has primarily been conducted through PGS or, more recently, PS. PS is provided in a private, confidential setting by an experienced practitioner with specialised supervision training. The Christchurch Radiation Oncology Centre trialled PS [11] and found that the twelve RTs who volunteered to receive PS reported improvements in career progression, job satisfaction, fulfilment, and improved reflective practice. This success led to the permanent implementation of PS within the department. One of the study's key findings was that some RTs, who had initially been considering leaving the profession, chose to stay, contributing to improved staff retention.

PGS is a peer‐led model [12] of supervision, where small groups share workplace challenges with similar individuals [13] in a safe environment [14]. However, for PGS to be effective, strict guidelines must be followed regarding the structure and process of the groups to prevent them from dissolving into unproductive grievance sharing [13]. PGS was trialled in five of the then nine RT centres across New Zealand in 2017 and 2018 [13]. This study found that while successful for newly qualified RTs, RTs with more years of experience found it difficult to adhere to the structure and processes of PGS, often using the groups more as a support network than a formal supervision platform. As a result, they did not fully obtain the intended benefits of group supervision. Following this study, third‐year RT students were introduced to PGS in 2019. A pilot study [15] of these students found they had improved self‐awareness and better stress management. As a result of these positive findings, the Bachelor of Radiation Therapy programme introduced mandatory PGS sessions for students on clinical placements to support their progress.

FGS integrates elements of PGS and PS, as it is conducted in small groups of professionals who meet with a trained supervisor to share experiences [8] and seek reflective practice through multiple viewpoints. Like PGS, FGS offers the benefits of peer learning, enhanced reflection, coping strategies, and well‐being. Often referred to as ‘reflective practice groups’ in nursing literature, FGS has been evaluated with positive outcomes for nurses in acute hospital settings [16] and cancer care nurses [9]. However, RTs have not engaged in FGS in New Zealand.

The Wellington Hospital Allied Health Workforce Development Team invited allied health staff from three disciplines—sonographers, allied health assistants, and radiation therapists—to trial FGS. These professions did not have supervision mandated as part of their clinical practice. Like other forms of supervision, FGS can provide a platform for participants to express their concerns, potentially improving job retention by offering a sense of being heard and contributing to identifying solutions [4]. This study focuses on the experiences of the RTs who participated in FGS. The aim of this study was to explore the RTs' perceptions of FGS and compare these with their experiences with any previous supervision they may have received.

## 2 Methodology

The University of Otago Human Ethics Committee granted ethical approval for this mixed‐method pilot study (reference number 24/0347), which included both qualitative and quantitative elements.

### 2.1 Participants

In February 2024, the Allied Health Workforce Coordinator facilitated an in‐service for RT staff at the Wellington Blood and Cancer Centre (WBCC). The in‐service session outlined the purpose of FGS, which motivated seven RTs (at the time, there were 30.4 FTE) to participate in the initiative, forming two groups. Like PGS, the groups were matched to years of experience, so one group had more experienced staff and the other the less experienced staff. This helps ensure that the groups' work experiences are more likely to be shared. These groups initially met in May 2024 and continued to meet approximately every four weeks over six months, with the final meeting held in November 2024. Meetings lasted 60–90 min, with the agenda being decided at the beginning of each session by the group members, and the facilitator deciding on what would be discussed and allocating time for discussion points. Every meeting was facilitated by a trained supervisor, an Allied Health professional who was external to the RT department. The facilitator completed an in‐house training on facilitating group supervision and was already experienced in both supervision and facilitating groups as part of their job role. The facilitator was the same for each session, which is recommended to maintain continuity [5]. The facilitator also maintained brief supervision records, which were securely stored and available to group members upon request, and were destroyed after the contract's termination.

### 2.2 The Questionnaire

After six months of routine FGS meetings, an RT independent of the researchers sent an email invitation to the seven RTs to complete a questionnaire (QUALTRICS) to evaluate their experience of FGS. This was sent two weeks after the last FGS session. The survey remained open for three weeks, and completing the survey indicated the participant's consent. Regular reminders were sent to the RTs, and all seven completed the questionnaire.

The questionnaire included the Clinical Supervision Evaluation Questionnaire (CSEQ) [17]. The CSEQ consists of 14 statements divided into three subscales: purpose (3 statements), process (5 statements), and impact (6 statements), which together address participants' perceptions of group supervision. Participants were required to indicate their opinion on a five‐point Likert scale, ranging from strongly agree to strongly disagree, with each statement being mandatory to score.

Two additional questions were included to assess whether the group supervision met participants' expectations and needs, along with three open‐ended questions allowing for more detailed feedback. These questions asked whether participants would choose to continue group supervision and whether they had participated in any type of group supervision before. They also provided a space for any further comments they wished to add. Finally, four demographic questions were included to gather information on participants' gender, ethnicity, age, and years of qualification.

### 2.3 Data Analysis

The collected data from the CSEQ and the thematic analysis of the open‐ended questions were analysed. The data will be stored electronically for 5 years.

The quantitative portion of the data was analysed by grading the responses on the CSEQ. A score of −2 corresponded with ‘strongly disagree’, −1 to ‘disagree’, 0 to ‘no opinion’, +1 to ‘agree’, and +2 to ‘strongly agree’ [18]. Based on this, the lowest possible score is −28, indicating strong disagreement, and the highest possible score is +28, indicating strong agreement. A score of less than 0 indicates a negative view of FGS. These scores and the mean score for each statement are displayed in Table 2.

The thematic analysis followed a systematic process using Braun and Clarke's six‐phase framework—familiarisation with data, generating initial codes, searching for themes, reviewing themes, defining and naming themes, and writing [20]. The analysis began with initial familiarisation with the data through repeated readings. This was followed by the identification of key terms and recurring patterns within the data, which were then coded [20]. From these, the key themes in the data were derived. To reduce bias, the analysis was initially performed independently by two of the researchers and later compared to reach a consensus on the final themes.

## 3 Results

All seven RTs completed the QUALTRICS questionnaire, achieving a 100% response rate. All participants were female, with three identifying as NZ European, and the remainder representing a range of ethnicities. Participants' ages ranged from 20 to over 28 years, with varying years of experience as qualified RTs (Table 1).

**TABLE 1:** Participant characteristics.

	No. of RTs (*n* = 7)
Gender	
Male	0
Female	7
Non‐binary/third gender	0
Prefer not to say	0
Other	0
Ethnicity	
NZ European	3
Māori	0
Samoan	0
Cook Island Māori	0
Chinese	1
Indian	1
Other	2
Age	
20–23 years	1
24–27 years	3
28+ years	3
Prefer not to say	0
Years qualified	
0–5 years	4
6+ years	3
Prefer not to say	0

### 3.1 CSEQ Results

Table 2 presents the CSEQ results on FGS's purpose, process, and impact. The total score represents the sum of all individual scores, while the mean score (MS) reflects the average rating for each statement, ranging from −2 to +2.

**TABLE 2:** Clinical supervision evaluation questionnaire (CSEQ) Results (*n* = 7).

Purpose	Strongly disagree	Disagree	No opinion	Agree	Strongly agree	Total score^a^	Mean score
The purpose of group supervision is to improve patient care (1)	0	−2^b^	0	+1	+8	+7	1
The purpose of group supervision is to enable RTs to feel confident in their own practice (9)	0	0	0	+2	+10	+12	1.71
I was clear about what I wanted to get out of group supervision (14)	0	0	0	+4	+6	+10	1.43

Process	Strongly disagree	Disagree	No opinion	Agree	Strongly agree	Score	Mean score
I felt safe sharing clinical issues in group supervision sessions (2)	0	0	0	+4	+6	+10	1.43
There were well‐established ground rules in my group (13)	0	0	0	+2	+10	+12	1.71
I believe that any confidences I shared were respected (3)	0	0	0	+1	+12	+13	1.86
There was mutual trust between the members in my group (5)	0	0	0	+2	+10	+12	1.71
I felt confident about bringing issues to group supervision (10)	0	0	0	+5	+4	+9	1.29

Impact	Strongly disagree	Disagree	No opinion	Agree	Strongly agree	Score	Mean score
Being part of group supervision helped to develop my self‐awareness (7)	0	0	0	+6	+2	+8	1.14
I have gained new clinical insights through group supervision (4)	0	−1	0	+3	+6	+8	1.14
Group supervision has made me more aware of areas of skill I need to improve (12)	0	0	0	+5	+2	+7	1
Group supervision has definitely had a positive impact on the quality of care I provide (6)	0	0	0	+4	+6	+10	1.43
Group supervision has helped me cope with any stresses at work I may have (11)	0	0	0	+5	+4	+9	1.29
Group supervision has helped me feel more confident about dealing with my job (8)	0	0	0	+5	+4	+9	1.29

^a^ Total score for each statement is calculated based on the RTs responses: strongly agree scores 2 (1 RT strongly agreeing = 2 points, 2 RTs strongly agreeing = 4 points), agree scores 1 (1 RT agreeing = 1 point), no opinion scores 0 (2 RTs with no opinion = 0), disagree scores −1 (1 RT disagreeing = −1), and strongly disagree scores −2 (1RT strongly disagreeing = −2) [15, 19].

^b^ In this case, there was: 2 RTs who disagreed, 1 RT who agreed, 4 RTs who strongly agreed. The total number of RTs is 7.

#### 3.1.1 Purpose

All participants agreed that the purpose of facilitated group supervision was to enable RTs to feel confident in their practice (MS 1.71), and all also agreed that they were clear about what they wanted from FGS (MS 1.43). Five RTs agreed that the purpose of group supervision was to improve patient care, while two disagreed.

#### 3.1.2 Process

All seven participants felt the group was a safe (MS 1.43) and confidential (MS 1.86) space with well‐established ground rules (MS 1.71). They all agreed there was mutual trust between members (MS 1.71) and felt confident in bringing issues to the group (MS 1.29).

#### 3.1.3 Impact

FGS was highly impactful, with six of the seven RTs reporting that they gained new insights, and all agreed they became more aware of skills they could improve (MS 1). Everyone felt they gained self‐awareness from FGS (MS 1.14) and that it would positively impact the quality of care provided (MS 1.43). They also agreed FGS had helped them cope with work‐related stress (MS 1.29) and felt more confident dealing with their job (MS 1.29).

### 3.2 Total Scores and Individual Scores

Six participants had a total CSEQ score greater than 14, indicating a positive overall result. One participant scored 12, still reflecting a largely positive perception of FGS. The total mean score for RTs with 0–5 years of qualification was 18.25, while the total mean score for those with more than 5 years of qualification was 21.

A total of 98 individual scores were recorded (14 CSEQ questionnaire questions × 7 participants = 98 individual scores). There were 49 ‘agree’ scores, 45 ‘strongly agree’ scores, 3 ‘disagree’ scores, and 1 ‘no opinion’ score. The statement with the most positive responses was Statement 3: “I believe that the confidences I shared were respected,” with six out of seven respondents (86%) strongly agreeing. The statement with the most disagreement was Statement 1: “The purpose of group supervision is to improve patient care,” with two respondents (29%) disagreeing.

### 3.3 Other Questions

Two questions were asked after the CSEQ, both using a Likert scale to score the answers. The first question asked whether FGS met their expectations, and the second asked whether it met their needs. Four participants (57.2%) agreed with both statements, and three participants (42.8%) strongly agreed.

### 3.4 Analysis of the Qualitative Data

All seven RTs completed the three open‐ended questions. These questions sought participants' additional comments, their willingness to continue with FGS, and a comparison of FGS with any previous supervision experiences they may have had. The results were largely positive, which was attributed to the facilitator leading the groups. However, two participants had not experienced any previous supervision, as it had not been offered to them, and could not provide a comparison. The other five RTs had previous experience with PGS; however, all five preferred FGS.

The group's answers revealed three main themes:

- The development of valuable insights and coping strategies.
- The environment enhanced safety and reflection.
- Discussing shared experiences reduced stress.

#### 3.4.1 The Development of Valuable Insights and Coping Strategies

A prominent theme across group responses was the high value placed on the presence of a facilitator, which allowed for the development of insights and coping strategies. The sessions had a clear structure and agenda (something that was often missing in PGS) and fostered active participation from all members, and encouraged the introduction of new perspectives on their challenges. Additionally, the facilitator offered external coping strategies, which participants reported as highly beneficial and superior to PGS.

“Having a facilitator creates a clear structure to every session, sessions are kept on track, and participants have a clear expectation of what is required of them in each session.” (0–5 years qualified)

“I think the major benefit is in the facilitation of it by someone separate from my practice; I found it helpful to have an outside perspective and someone to keep us on track in our reflections and questions.” (5+ years qualified)

“In supervision having a person/facilitator who is independent to listen and provide guidance was far more constructive. We left this session with tools or things that we could do in challenging situations” (5+ years qualified)

The RTs also emphasised that the structure provided by the facilitator was particularly valuable, given the high‐pressure environment of the department, where they regularly experience stress and heavy workloads. Participants described this structure as instrumental in reducing stress during the sessions, as the facilitator guided the discussions and provided a clear agenda, which helped manage the flow and focus of the meetings. This structure allowed the RTs to engage more effectively and feel supported in addressing their challenges.

“During my previous experience of non‐facilitated group supervision, a lot relied on group members actively participating in every session. In the current work environment of short staffing and high workload, this would take a lot of energy from participants. Having a facilitator creates a clear structure to every session, sessions are kept on track, and participants have a clear expectation of what is required of them in each session. This takes away some of the effort required by participants in a non‐facilitated session.” (5+ years qualified)

#### 3.4.2 The Environment Enhanced Safety and Reflection

The RT's highly valued the safe and confidential space the facilitator created, allowing them to openly share their stressors. Participants reported that the facilitator actively encouraged participation from all group members, creating an environment of mutual support and trust. This collaborative approach contributed to developing a safe, communal space where individuals felt comfortable discussing challenges and concerns without fear of judgement.

“I found that the group supervision was a safe platform to share and discuss any concerns or thoughts I had in both my work and personal life.” (5+ years qualified)

“I think it's beneficial to have an open and honest and caring space to voice scenarios or thoughts. It is a safe space where people are able to bring anything and everything to discuss, and it's really powerful in that sense.” (0–5 years qualified)

#### 3.4.3 Discussing Shared Experiences Reduced Stress

The RTs highlighted the benefit of sharing their challenges with a group of individuals who understood their specific concerns. They noted that this shared understanding provided new perspectives on the situations they struggled with, facilitating deeper conversations. The group's ability to empathise and offer knowledgeable guidance based on their own experiences was a valuable resource for problem‐solving and personal growth.

“It was a great space to discuss any stressors confidentially and helped us gain insight on ways to manage with others who understand as they are in the same role.” (0–5 years)

“Supervision helped me gain perspective on issues I've dealt with at work and allowed me an opportunity to reflect on my practice.” (5+ years)

“The facilitator and my peers were able to provide insight, share their experiences of similar situations and reassure me that what I was feeling was normal.” (5+ years)

## 4 Discussion

This pilot study explored an area of supervised practice not previously investigated for New Zealand RTs and found that participants were highly receptive to FGS. All seven RTs expressed willingness to continue with FGS if it were offered more widely, viewing it as a beneficial initiative. Both quantitative and qualitative findings demonstrated a clear positive perception of FGS. Quantitative results indicated that participants experienced FGS as a safe, trustworthy, and well‐structured environment. The qualitative data reinforced this, highlighting safety within the group as a crucial factor supporting reflective practice.

Participants emphasised the significant value of having a facilitator. They noted that the facilitator provided structure and guidance—elements that previous studies on group supervision have often identified as lacking [12, 13]. A New Zealand national study on PGS reported similar benefits, with RTs appreciating the support of a group that could ‘understand’ their experiences and provide a safe space for reflection [13]. However, that study also found that the absence of a facilitator led to unclear expectations, which diminished the effectiveness of sessions. Notably, RTs with more than five years' experience were more likely to engage in informal, unstructured meetings [15].

In contrast, the current study on FGS found that RTs with over five years of experience achieved higher mean scores on the CSEQ than their less experienced colleagues. They also left comments emphasising the importance of the facilitator in fostering an effective and structured group environment. These findings suggest that, for more experienced staff, a skilled facilitator is central to the success of supervision sessions, with facilitated models offering the most significant benefit.

When asked to compare FGS with their previous supervision experiences, five RTs reported prior involvement in PGS, including the three RTs with less than five years of experience. These five participants described PGS as less productive and more difficult and stressful to navigate. Previous studies [13, 15] have shown that less experienced staff benefit from PGS when introduced early in their careers. In New Zealand, this approach is extended to student RTs, who receive PGS during their training. However, in this study, they preferred FGS and felt they gained more from a facilitated session.

The findings from RTs at the Christchurch Radiation Oncology Centre on professional supervision [11] align with this study, highlighting the supervisor as a crucial component in the success of one‐on‐one and group supervision sessions. Several benefits were associated with the presence of a professional supervisor, including enhanced self‐awareness, improved practice, and greater overall well‐being—all of which contributed to staff retention in radiation therapy. Notably, the PS study found that participants who had been considering leaving the profession were instead motivated to stay and invest further in their careers. The success of these sessions was shown to depend on the mutual effort invested by both the supervisor/facilitator and the supervisee.

Participants in the present FGS study also reiterated the importance of a skilled and committed facilitator, emphasising that the success of the sessions was primarily attributable to the quality of the supervisory relationship. They further noted that any new supervisor would need to be well‐trained and equally dedicated to achieving the same positive outcomes. Although the studies differed in that one was one‐on‐one, and the other was group supervision, the presence of an experienced supervisor contributed to the success of both. The effectiveness of both studies, with minimal negative feedback from participants, suggests that facilitated supervision is a promising option for supporting RTs in the future. PS has already been implemented permanently in the Christchurch Radiation Oncology Centre. The findings of this study have prompted the Wellington Blood and Cancer Centre to extend FGS to additional RTs who are keen to participate, with an intent to make FGS a permanent option for RTs in the future. The Allied Professions Workforce Development Team also plans to train more facilitators to support this expansion.

Prior research has examined the impact of facilitated group supervision (FGS) on the quality of life of Australian nurses, referring to FGS as reflective practice groups (RPG) in their analyses [16, 19, 21, 22]. Nurses experience stressors similar to those faced by RTs, such as high task demands and emotional labour [21, 22]. The findings from these nursing studies suggest that RPGs are associated with positive outcomes, including increased compassion satisfaction, enhanced group cohesiveness, and improved tolerance to uncertainty. RPG conducted over an extended period revealed a strong correlation between higher attendance rates in RPGs and better clinical outcomes [19].

Similar results have been reported by allied health professionals in New Zealand and Australia, concluding that FGS demonstrated positive impacts on confidence and the ability to perform daily patient interactions [23, 24]. A scoping review involved five groups: psychology, speech and language therapy, occupational therapy, physiotherapy, and social work. Overall, the results were positive, with staff respecting the dedicated safe space to reflect [23]. This study highlighted the importance and challenges of using the right supervision tools in Allied Health. Finding the right framework for RTs has been a challenge, too; what works in one centre may not work in another. The PS experience in the Christchurch Radiation Oncology Centre reported similar benefits for RTs [11], suggesting that extending the FGS programme over a longer period could yield comparable outcomes and better support job retention for RTs. A longitudinal study would be beneficial to examine the sustainability of these effects over time.

### 4.1 Implementing FGS Into RT Practice

To implement FGS at the Wellington Blood and Cancer Centre, the group facilitators completed an in‐house training programme for Facilitating Group Supervision and should have experience in both receiving and providing professional supervision. To increase psychological safety in the groups, someone external to the department should be utilised as the facilitator. The Allied Health Workforce Group provided the facilitators as part of their professional practice, so there was no financial cost to the cancer centre.

Timing of the sessions is 60–90 min per month depending on group size, optimally 4, maximally 6. Costs are time off the floor, but this is counteracted by an improvement in job satisfaction and a trend of less burnout, therefore, the potential for less staff attrition.

As of October 2025, the Wellington Centre now has four FGS sessions running for RT staff. It is voluntary for RTs to opt in to FGS. It can't be made mandatory as it is not mandated by the New Zealand Medical Radiation Technologists Board for RT to undergo supervision [25].

### 4.2 Limitations

The limitations of this study include the small, all‐female sample, which limits the generalisability of the findings. The study's six‐month duration restricts the ability to assess long‐term outcomes. Additionally, the time gap between the completion of the final FGS session and the survey distribution may have introduced recall bias.

## 5 Conclusion

This pilot study investigated radiation therapists' perceptions of facilitated group supervision and compared them to their experiences with previous supervision formats. The findings suggest that FGS positively impacted all participants, particularly more experienced RTs, who reported greater benefits compared to their experience with PGS. The structured, guided environment was perceived as a safe space for discussing workplace stressors and enhancing reflective practice. These results highlight the value of a well‐organised and professionally facilitated supervision framework, particularly for experienced practitioners.

While the small sample size limits the generalisability of the findings, the study offers meaningful insights into the role of structured supervision in supporting professional development within the RT workforce. Further research is recommended to evaluate FGS's effectiveness more broadly and assess the long‐term impact of FGS on professional growth and staff retention. Overall, this study contributes to the growing body of evidence endorsing tailored supervisory models for RTs and underscores their potential to enhance job satisfaction and improve staff retention rates.

## Conflicts of Interest

The authors declare no conflicts of interest.

## Data Availability

The data that support the findings of this study are available from the corresponding author upon reasonable request.

## References

[1] G. K. B. Halkett , J. McKay , D. G. Hegney , et al., “Radiation Therapists' and Radiation Oncology Medical Physicists' Perceptions of Work and the Working Environment in Australia: A Qualitative Study,” European Journal of Cancer Care 26 (2017): e12511. 10.1111/ecc.12511 27147506

[2] G. K. B. Halkett , M. N. Berg , L. J. Breen , et al., “Sustainability of the Australian Radiation Oncology Workforce: A Survey of Radiation Therapists and Radiation Oncology Medical Physicists,” European Journal of Cancer Care 27 (2018): e12804. 29341295 10.1111/ecc.12804

[3] M. R. Taylor and J. G. Oetzel , “The Sustainability of the New Zealand Radiation Therapy Workforce: Factors That Influence Intent to Leave the Workplace and Profession,” Technical Innovations & Patient Support in Radiation Oncology 16 (2020): 77–82. 33319075 10.1016/j.tipsro.2020.11.002 PMC7724194

[4] S. M. Kilminster and B. C. Jolly , “Effective Supervision in Clinical Practice Settings: A Literature Review,” Medical Education 34 (2000): 827–840. 11012933 10.1046/j.1365-2923.2000.00758.x

[5] A. L. Valentino , L. A. LeBlanc , and T. P. Sellers , “The Benefits of Group Supervision and a Recommended Structure for Implementation,” Behavior Analysis in Practice 9 (2016): 320–328. 27920963 10.1007/s40617-016-0138-8 PMC5118257

[6] D. Ray and M. Altekruse , “Effectiveness of Group Supervision Versus Combined Group and Individual Supervision,” Counsellor Education and Supervision 40 (2000): 19–30.

[7] M. M. Saab , C. Kilty , E. Meehan , et al., “Peer Group Clinical Supervision: Qualitative Perspectives From Nurse Supervisees, Managers, and Supervisors,” Collegian 28 (2021): 359–368.

[8] M. O'Keeffe and F. James , “Facilitated Group Supervision: Harnessing the Power of Peers,” Journal of Paediatrics and Child Health 50 (2014): 944–948. 24957327 10.1111/jpc.12638

[9] J. McVey and T. Jones , “Assessing the Value of Facilitated Reflective Practice Groups,” Cancer Nursing Practice 11 (2012): 32–37.

[10] S. Karvinen‐Niinikoski , L. Beddoe , G. Ruch , and M. Tsui , “Professional Supervision and Professional Autonomy,” Aotearoa New Zealand Social Work 31 (2019): 87–96.

[11] G. Dungey , S. Thomson , and P. R. Lopez , “Radiation Therapists' Perceptions of Participating in Professional Supervision—A Pilot Study,” Journal of Medical Radiation Sciences 72 (2024): 54–62, 10.1002/jmrs.822. 39245909 PMC11909698

[12] T. Tulleners , C. Campbell , and M. Taylor , “The Experience of Nurses Participating in Peer Group Supervision: A Qualitative Systematic Review,” Nurse Education in Practice 69 (2023): 103606. 36989698 10.1016/j.nepr.2023.103606

[13] G. Dungey , H. Neser , and D. Sim , “New Zealand Radiation Therapists' Perceptions of Peer Group Supervision as a Tool to Reduce Burnout Symptoms in the Clinical Setting,” Journal of Medical Radiation Sciences 67 (2020): 225–232. 32431058 10.1002/jmrs.398 PMC7476202

[14] O. Doody , C. O'Donnell , L. Murphy , J. Turner , and K. Markey , “The Establishment and Value of Peer Group Clinical Supervision: A Qualitative Study of Stakeholders' Perspectives,” Journal of Clinical Nursing 33 (2024): 4061–4076. 38837472 10.1111/jocn.17315

[15] G. M. Dungey and P. H. Bates , “Radiation Therapy Students' Perceptions of Peer Group Supervision: A Pilot Study,” Journal of Medical Radiation Sciences 68 (2021): 426–434. 34263548 10.1002/jmrs.527 PMC8656198

[16] B. R. Davey , S. J. Byrne , P. M. Millear , C. Dawber , and L. Medoro , “Evaluating the Impact of Reflective Practice Groups for Nurses in an Acute Hospital Setting,” Australian Journal of Advanced Nursing 38 (2021): 6–17.

[17] S. Horton , M. de Lourdes Drachler , A. Fuller , and J. C. de Carvalho Leite , “Development and Preliminary Validation of a Measure for Assessing Staff Perspectives on the Quality of Clinical Group Supervision,” International Journal of Language & Communication Disorders 43 (2008): 126–134. 17852531 10.1080/13682820701380031

[18] A. Joshi , S. Kale , S. Chandel , and D. Pal , “Likert Scale: Explored and Explained,” British Journal of Applied Science & Technology 7 (2015): 396–403.

[19] C. Dawber and T. O'Brien , “A Longitudinal Comparative Evaluation of Reflective Practice Groups for Nurses Working in Intensive Care and Oncology,” Journal of Nursing Care 2 (2014): 133.

[20] V. Braun and V. Clarke , “Using Thematic Analysis in Psychology,” Qualitative Research in Psychology 3, no. 2 (2006): 77–101.

[21] N. Golfenshtein and A. Drach‐Zahavy , “An Attribution Theory Perspective on Emotional Labour in Nurse–Patient Encounters: A Nested Cross‐Sectional Study in Paediatric Settings,” Journal of Advanced Nursing 71 (2015): 1123–1134. 25558788 10.1111/jan.12612

[22] M. K. Sundgren , C. Dawber , P. M. Millear , and L. Medoro , “Reflective Practice Groups and Nurse Professional Quality of Life,” Australian Journal of Advanced Nursing 38 (2021): 49–67.

[23] S. McGuinness and S. Guerin , “Interprofessional Supervision Among Allied Health Professionals: A Systematic Scoping Review,” Journal of Interprofessional Care 38 (2024): 739–758. 38678372 10.1080/13561820.2024.2343837

[24] A. Davis , N. Meloncelli , A. Hannigan , and W. Ward , “Evaluation of a Model of Online, Facilitated, Peer Group Supervision for Dietitians Working in Eating Disorders,” Journal of Eating Disorders 10 (2022): 93. 35787290 10.1186/s40337-022-00617-7 PMC9252553

[25] New Zealand, Medical Radiation Technologists Board (NZMRTB) , “Competence Standards for Medical Imaging and Radiation Therapy Practitioners in Aotearoa New Zealand. Version 3,” (2023).

